# Role of microRNA Shuttled in Small Extracellular Vesicles Derived From Mesenchymal Stem/Stromal Cells for Osteoarticular Disease Treatment

**DOI:** 10.3389/fimmu.2021.768771

**Published:** 2021-11-01

**Authors:** Eliana Lara-Barba, María Jesús Araya, Charlotte Nicole Hill, Felipe A. Bustamante-Barrientos, Alexander Ortloff, Cynthia García, Felipe Galvez-Jiron, Carolina Pradenas, Noymar Luque-Campos, Gabriela Maita, Roberto Elizondo-Vega, Farida Djouad, Ana María Vega-Letter, Patricia Luz-Crawford

**Affiliations:** ^1^ Laboratorio de Inmunología Celular y Molecular, Centro de Investigación Biomédica, Facultad de Medicina, Universidad de Los Andes, Santiago, Chile; ^2^ Department of Physiology, Pontificia Universidad Católica de Chile, Santiago, Chile; ^3^ Facultad de Ciencias Biológicas, Millennium Institute for Immunology and Immunotherapy, Santiago, Chile; ^4^ Departamento de Ciencias Veterinarias y Salud Pública, Facultad de Recursos Naturales, Universidad Católica de Temuco, Temuco, Chile; ^5^ Laboratorio de Biología Celular, Departamento de Biología Celular, Facultad de Ciencias Biológicas, Universidad de Concepción, Concepción, Chile; ^6^ Institute for Regenerative Medicine and Biotherapy (IRMB), Univ Montpellier, Institut national de la santé et de la recherche médicale (INSERM), Montpellier, France; ^7^ IMPACT, Center of Interventional Medicine for Precision and Advanced Cellular Therapy, Santiago, Chile

**Keywords:** microRNA, small extracellular vesicles, mesenchymal stem cells, osteoarthritis, rheumatoid arthritis

## Abstract

Osteoarticular diseases (OD), such as rheumatoid arthritis (RA) and osteoarthritis (OA) are chronic autoimmune/inflammatory and age-related diseases that affect the joints and other organs for which the current therapies are not effective. Cell therapy using mesenchymal stem/stromal cells (MSCs) is an alternative treatment due to their immunomodulatory and tissue differentiation capacity. Several experimental studies in numerous diseases have demonstrated the MSCs’ therapeutic effects. However, MSCs have shown heterogeneity, instability of stemness and differentiation capacities, limited homing ability, and various adverse responses such as abnormal differentiation and tumor formation. Recently, acellular therapy based on MSC secreted factors has raised the attention of several studies. It has been shown that molecules embedded in extracellular vesicles (EVs) derived from MSCs, particularly those from the small fraction enriched in exosomes (sEVs), effectively mimic their impact in target cells. The biological effects of sEVs critically depend on their cargo, where sEVs-embedded microRNAs (miRNAs) are particularly relevant due to their crucial role in gene expression regulation. Therefore, in this review, we will focus on the effect of sEVs derived from MSCs and their miRNA cargo on target cells associated with the pathology of RA and OA and their potential therapeutic impact.

## Introduction

An excessively prolonged imbalance of the immune system response can lead to a vast array of inflammatory and autoimmune disorders. Moreover, genetic predisposition and epigenetic regulations, including environmental factors and age, promote autoimmune, inflammatory, and degenerative diseases development (1). These illnesses imply a high economic burden for the healthcare system and those who suffer from them (2, 3). Osteoarticular diseases (OD), such as osteoarthritis (OA), and rheumatoid arthritis (RA), have raised particular concern in the last decades due to the increase of medical consults. They affect roughly 23% of the population over 40 worldwide for knee OA (the most common articulation affected by OA) (4, 5), and around 0.5% of the worldwide population for RA (6). Moreover, both OA and RA cause a great deal of pain and discomfort to the patients, impacting their quality of life (7). Without a cure for OD, patients rely mainly on non-steroidal anti-inflammatory drugs (NSAIDs), analgesics, and glucocorticoids as the primary options to manage the symptoms (8, 9). Unfortunately, these treatments lack disease- and structural-modifying capabilities and even worse, their prolonged use is associated with severe side effects (9, 10).

Thus, alternative therapies are still needed to treat autoimmune/inflammatory and degenerative diseases like OA and RA. Both diseases are mainly defined by the loss of articular cartilage and are known to affect people of all races, genders, and ages (11, 12). Numerous therapeutic efforts have been made to restore the affected joints, including tissue engineering to promote tissue regeneration. Recently, cell-based therapies have had a considerable rise, such as the regulatory T cell therapy. However, their high cost and the technical difficulties in producing off-the-counter cell therapies remain significant hurdles for their clinical application (13). Three types of cell treatment are used in clinical trials for OA or degenerative environments; articular chondrocytes, meniscal fibrochondrocytes, and mesenchymal stem/stromal cells (MSCs), where the latter has shown encouraging results (11, 14–17). MSCs are multipotent stem cells of mesodermal origin that can be defined as a cell population with the hallmark self-renewal properties and differentiation into chondrogenic, osteogenic, and adipogenic lineages (18). Although therapy using MSCs has achieved significant progress, stem cell-based therapies have not fulfilled the initial promise. Some remaining drawbacks include the inconveniences associated with high costs and potential side effects, leading to inconsistency among preclinical and clinical trials (19).

In recent years, the therapeutic benefit of MSCs has been attributed to their functions through cell–to–cell contact and, more prominently, paracrine communication. The main mediators of paracrine communication are small extracellular vesicles (sEVs), which play an essential role as an alternative mechanism by which MSCs regulate different biological processes (20, 21). sEVs are heterogeneous particles that are delimited by a lipid bilayer membrane, whose primary function is to act as vehicles of cellular communication, transporting and transferring several bioactive molecules, such as proteins, peptides, lipids, messenger RNA (mRNA), and microRNA (miRNA) (22). miRNAs are small 20–22-nucleotide-long non-coding RNAs, which mediate post-transcriptional gene silencing by binding to the 3’-untranslated region (UTR) or open reading frame (ORF) region of target mRNAs (23) unpairing protein translation and causing a rapid tuning of cell fate decisions in response to environmental cues (24). Although sEVs can carry different types of cargo, increasing evidence points at miRNAs as significant mediators for the effects of these vesicles over the target cells (25, 26). Noteworthy, miRNAs regulate the immune system and signaling pathways related to extracellular matrix synthesis, chondrocyte survival, and proliferation (27–29). In addition, the auspicious use of sEVs as “cell-free cellular therapies’’ provides substantial advantages in contrast to whole-cell therapy, such as their easy handling and minimizing the risks of rejection (30). This review summarizes the current knowledge of MSC derived sEVs (MSC-sEVs) and their miRNA cargo as a potential and attractive substitute for treating autoimmune/inflammatory and degenerative disorders.

## MSC-Based Therapy For OD Treatment

MSCs are multipotent fibroblast-like cells of mesodermal origin that have been described in several mammals, including humans and mice (31). According to the International Society of Cell Therapy (ISCT), three major criteria define MSCs: their capacity to adhere to plastic surfaces under culture conditions (32), their ability to self-renew and differentiate toward mesodermal lineages, such as adipogenic, chondrogenic and osteogenic (33) lineages, as well as the expression of surface markers CD105, CD73, and CD90 in the absence of hematopoietic markers including CD45, CD34, CD14 or CD11b, CD19, and HLA‐DR (18, 34). These cells are found in various tissues, including bone marrow, adipose tissue, dental pulp, endometrium, amniotic fluid, placenta, and umbilical cord, among others (35). However, bone marrow and adipose tissues represent the most common sources for MSCs isolation because of their availability (36–47).

MSCs display a wide variety of biological functions, such as secretory (48), immunomodulatory (49) and homing (50) properties, representing a stem cell population with demonstrable progenitor cell functionality (33, 51) and a promising candidate for cell-based therapies. Illustrating this, ClinicalTrial.gov (https://clinicaltrials.gov/) lists 10406 phase I or II trials using MSCs in skin, bone, cartilage, heart, kidney, lung, liver, diabetes, immune/autoimmune diseases and even for COVID-19. Among these trials, 222 registered studies are using MSCs for OA and 55 for RA. OD are well–documented candidates for MSC treatment. Recent studies have shown that OA patients treated with an intra-articular injection of MSCs display a substantial enhancement in cartilage coverage and quality, relieving pain, ameliorating disability, and significantly improving their quality of life (11, 12, 52, 53). Similarly, a phase Ia clinical trial in RA demonstrated the reduction of pro-inflammatory cytokines in patients injected with MSCs and revealed no short-term safety concerns (54). This data supports the potential of MSCs as an effective treatment for OA and RA patients.

Several studies have shown that MSCs can replace several damaged tissues *in vivo*. Mirza and collaborators showed that undifferentiated MSCs seeded on a graft were able to grow and restore a thick multicellular layer mimicking mature vascular tissue (55), whereas Sheng and collaborators were able to successfully transplant MSCs and regenerating sweat glands in patients *in vivo* (55, 56). Previous studies have demonstrated that MSCs can regulate the inflammatory response by suppressing mononuclear cells and promoting anti-inflammatory subsets from innate and adaptive immunity, including T-cells (57, 58). It has been well described that MSCs regulate T-cells activation and proliferation without the need for the cell to cell contact, suggesting the involvement of secreted soluble factors as the mechanism of action (59, 60). Additionally, MSCs negatively regulate natural killer cells (NK) activity, dendritic cells (DC) maturation, and B-cells proliferation while promoting Treg induction [Reviewed in (61, 62)]. It has also been shown that one of the hallmarks of MSC therapeutic potential is the regulation of cytokine production, including IFN-γ, TNF-α, and IL-10 (62). By modulating different immune cells involved in autoimmune diseases’ pathogenesis, MSCs have a promising therapeutic potential. Although some mechanisms require the cell to cell contact, MSCs secretome seems to mediate most of their therapeutic effects in several pathologies, including OD (63, 64).

In the last few years, several studies suggest that MSC therapies in clinical applications do not show severe adverse effects showing promising therapeutic benefits (65). Nonetheless, the clinical application of MSCs and the fast development of commercial products show contradicting outcomes in clinical application and unsatisfactory therapeutic effects, primarily due to their low survival and homing capacity *in vivo* (19). Site-specific injection seems to be better to obtain more efficiency results [Reviewed in (66, 67)]. Therefore, to use MSCs as a successful treatment, these difficulties must be overcome. The most critical challenges are donor heterogeneity, stemness stability and differentiation capacities, limited expansion capacities, homing capacity, and rejection risks (68). In this regard, their derivatives including extracellular vesicles come as a promising solution as a cell-free based therapy due to their role as molecule delivery vehicles that mimic the effects of the parent on the target cell (66).

## MSC-Derived Small Extracellular Vesicles as Therapeutic Tools to Treat Osteoarticular Diseases

Extracellular vesicles (EVs) are membrane-bound nanostructures released that act as essential mediators of cell-to-cell communication under physiological and pathological conditions (69). According to their size, EVs can be classified as apoptotic bodies (more than 1000nm), microvesicles (between 40-1000nm), and exosomes (50-200nm) (70). EVs can be generated directly by budding from the plasma membrane (microvesicles) or after fusion of multivesicular bodies (related to the endocytic pathway) with the plasma membrane to release intraluminal vesicles (exosomes). EVs are normally obtained by differential centrifugation protocols and the exosome enriched fraction also contains small microvesicles (smVs) commonly referred to as small extracellular vesicles (sEVs) (71, 72). sEVs can be further characterized by the expression of exosome-associated markers such as TSG101, ALIX, and tetraspanin proteins such as CD9, CD63 or CD81 (70). Released sEVs can either be readily taken up by neighboring or by distant cells due to their ability to travel through body fluids and mimic the parent cell’s effect on the target cell (70). Due to the natural role of sEVs in cell-to-cell communication, they are readily taken up through phagocytosis, micropinocytosis, and endocytosis mediated by lipid raft, caveolin or clathrin (73, 74). Although sEVs can be delivered to any cell type, they are internalized in a highly cell type-specific manner that depends on recognizing typical sEV surface molecules by the cell or tissue, making them ideal therapeutic delivery systems [Reviewed in (74)].

A substantial advantage of using sEVs as therapeutic carriers is that they are nearly non-immunogenic and are capable of homing to distant tissues where the inflammation is located (75, 76) Indeed, mice injected with both wild-type and engineered sEVs showed no toxicity nor a significant immune response, further adding to the safety of sEV based therapies (77). However, the delivery and the frequency of sEVs injection on patients still needs to be addressed, in order to determine the most efficient strategy to obtain positive clinical outcomes.

In preclinical models, it has been described that MSCs-sEVs inhibit TNF-α induced collagenase activity and promote cartilage regeneration in chondrocytes derived from OA patients *in vitro* (75, 76). Moreover, MSCs-sEVs significantly improve OA progression by inhibiting cartilage degeneration in the collagenase-induced OA murine model (78). MSCs-sEVs were also shown to enhance the production of immature DCs that secrete IL-10, which are involved in suppressing inflammatory T-cell responses (76, 79, 80). On the other hand, Zhu and colleagues demonstrated that sEVs could reduce arthritis index, leukocyte infiltration, and, most importantly, destruction of the joint in a CIA mice model. These sEVs lowered Th1 and Th17 cells’ frequencies through miRNA targeting of STAT3 and T-bet, having a potential role in treating arthritis (81). Munir and colleagues also proved that treating CIA in mice with MSCs decreased the severity of the disease by dampening the pathogenic immune response. Mice that received this treatment had reduced IL-6 and TNF-α, increased IL-10 in their joints and increased the frequency of Tregs in their spleen and lymph nodes, and a lower Th1:Th17 ratio (66). Other studies have demonstrated that sEVs can decrease the clinical signs of inflammation present in the CIA model by polarizing B lymphocytes into Breg-like cells (82). Therefore, evidence supports the repairing properties of MSCs-sEVs in joint tissue, especially after intra-articular administration (83). These and other preclinical studies of MSCs-sEVs show that these potential treatments are safe and scalable for clinical application (20).

Since phase III clinical trials have shown inconsistent results in RA and OA without cartilage regeneration despite the promising preclinical studies (52, 84), their derived sEVs could also display conflicting results for RA and OA treatment. Several techniques to improve MSCs therapy have been recommended to overcome these issues [Reviewed in (85)]. For example, hypoxia preconditioning and 3D culture can increase the production of pro-chondrogenic factors (86). Additionally, sEVs action can be strengthened by modifying their specific cargo (87, 88), or by treatment with immunosuppressive cytokines, such as IL-10 (89), enhancing their anti-inflammatory and chondroprotective properties. Moreover, it has been shown that the genetic engineering of MSCs affects their derived sEVs, improving their immunosuppressive and chondroprotective abilities (87), where sEVs demonstrated to enhance chondrogenesis and suppress cartilage degradation (88).

The therapeutic effect of sEVs in the target cell is directly dependent on their cargo, which can be composed of a wide variety of molecules, including proteins, peptides, lipids, and several nucleic acids such as DNA, messenger RNA and microRNAs [Reviewed in (21)]. Although the effects of other sEV cargos cannot be excluded, proteins and miRNAs are considered the main mediators of the effect of sEVs in target cells. Proteomic analysis in sEVs has identified thousand proteins implicated in key biological processes such as sEV biogenesis, cellular structure, tissue repair and regeneration, and inflammatory response [Reviewed in (90)] Indeed, Chaubey and collaborators, validated TSG-6 as one of the protein mediators of MSC-sEV for immunomodulation by inducing a decrease in neutrophil infiltration in a murine model of hyperoxia-induced lung injury (91). However, to determine the role of proteins and miRNA in mediating the therapeutic efficacy of sEVs, a relation between the concentration of miRNA and proteins in their cargo is needed (92). Moreover, it is not well defined whether proteins and miRNAs work independently or synergistically in target cells, indicating that further studies are needed in this field. On the other hand, miRNAs encompass an important fraction of the exosome content and arise as the main regulators of MSC-sEVs function (26, 93). miRNAs are small non-coding RNA highly conserved among species, which control gene expression through its binding capacity to the three prime untranslated region (3’-UTR) of the targeted mRNAs, for repressing the expression of the corresponding gene at a post-transcriptional level (94). Compared with transcriptional and epigenetic regulation, post-transcriptional processes are fast and therefore can instantly tune cell fate decisions in response to environmental cues (94). Moreover, miRNAs contained in sEV are protected from RNAse degradation and through their integrins and opsonins the delivery of their internal content is efficient (24). Indeed, Neviani and collaborators demonstrated that sEVs derived from inactivated natural killer (NK) cells showed an equal cytotoxic activity when compared to sEVs derived from activated NK cells. Indeed, inactivated NK derived sEVs showed low levels of killer proteins in their cargo (perforin 1, granzyme A, granzyme B) while still retaining their cytotoxic activity, showing that the protein cargo is not the main bioactive mediator (95). In line with these results, RNA-depleted sEVs lose their immunosuppressive activity on T-cells, demonstrating their pivotal role on MSC-sEVs immunoregulation.

## Relevance of miRNA in the Pathogenesis of OD

miRNAs are critical regulators in maintaining a healthy joint as they participate in chondrocyte homeostasis and in the regulation of inflammatory mediators (96, 97). Proof of this is the phenotype observed in Dicer (a key enzyme in the miRNA biosynthesis pathways) knock-out mice, whose growth plates exhibited a reduction in proliferating chondrocytes and accelerated differentiation into a hypertrophic type, resulting in severe skeletal growth defects and premature death (98). Accordingly, an imbalance of some miRNAs has been associated with OD in both human and murine models. Illustrating this, a study using the serum transfer mouse model of RA in C57BL/6 mice identified a total of 536 upregulated genes and 417 downregulated genes that are predicted targets of miRNAs with reciprocal expression in arthritic mice (99). Twenty-two miRNAs whose expression was most significantly changed between nonarthritic and arthritic mice regulated the expression of proteins involved in bone formation, specifically Wnt and BMP signaling pathway components.

While activation of canonical Wnt signaling promotes bone formation (100), Wnt signaling antagonists such as Dkk inhibit this pathway and have been shown to regulate the erosive process in RA (101, 102). Among the most upregulated miRNAs found by Maeda and colleagues was miR-221-3p, which is induced in the TNF-driven model of arthritis and fibroblast-like synoviocytes (FLS) from RA patients (103). In bone, synovium-derived miRNAs, including miR-221-3p, may control skeletal pathways that inhibit osteoblast differentiation from augmenting bone erosion in RA by regulating Dkk2. Similar studies in OA patients have revealed significant miRNA imbalance in cartilage, synovial fluid, and plasma (104). Several studies have shown that there is differential expression of several miRNAs in OA *versus* a healthy joint. By evaluating the expression of 365 miRNA in OA patients versus healthy donors, Iliopoulos and colleagues found 16 altered miRNA, providing one of the earliest insights on the osteoarthritic chondrocytes miRNA signature (105). A subsequent study showed that a set of 17 miRNA that contribute to cartilage remodeling presented an altered expression and suggested that these changes were due to epigenetic regulation (106). Murata and colleagues investigated whether, in plasma and synovial fluid, miRNA could be used as possible biomarkers for RA and OA, finding that some miRNAs can effectively differentiate between both diseases (107). Interestingly, 12 miRNA were overexpressed under the OA condition, all targeting important genes in chondrocyte maintenance and differentiation such as SMAD1, IL-1B, COL3A, VEGFA, and FGFR1 (104). Other reports point out imbalances in miRNAs associated with the regulation of ECM degradation enzymes. For example, the increase of miR-146a/miR-145/miR-22 and the decrease in miR-149/miR-125b/miR-558 causes ECM degradation. Some miRNAs such as miR-27b, miR-140, and miR-320 have been reported to target MMP13, a regulator of tissue repair and remodeling (108–110), while miR-92a-3p and miR-27b regulate ADAMTS expression, an enzyme that plays an important function in the degeneration of cartilage in RA and OA (111). Furthermore, it has been shown that the down-regulation of miR-140 inhibits IL-1β by inducing ADAMTS expression and that miR-27b regulates MMP-13 expression in human chondrocytes. Importantly, miR-27b, miR-140, and miR-146a are dysregulated in OA patients, suggesting a role for them in OA pathogenesis (108, 112, 113).

It has been widely reported that TGF-βs and BMPs regulate postnatal joint cartilage homeostasis and that dysregulated TGF-β and BMP signaling are often associated with OD [Reviewed in (114)]. These TGF-β superfamily members bind to the heteromeric receptor complex, comprised type I and II receptors at the cell surface, that transduce intracellular signals by activating Smad complex or mitogen-activated protein kinase (MAPK) cascade. BMPs have a chondroprotective role in different animal models of RA (115); specifically, it has been suggested that endogenous expression of BMPs is required to maintain chondrocytes phenotype *in vitro* (116, 117). However, its dynamic regulation has been observed in the CIA murine model, supporting a role for this pathway in RA (118). During CIA, BMP-2 and BMP-7 are upregulated in a TNF-dependent manner, a phenomenon accompanied by an increase in Smad-5 phosphorylation: thus, there is an increase in BMP signaling activity. Similarly, in an OA rat model, it was shown that IL1b upregulated BMP-2 through the MEK/ERK/Sp1 signaling pathways and that the administration of the BMP antagonist Noggin prevented cartilage degeneration and OA development (119). An observational study in OA patients showed that the levels miR-22, which targets BMP2, are increased in the progression of the disease (120). Furthermore, the inhibition of miR-22 has been shown to prevent inflammatory activity (105, 121). On the contrary to miR-22, miR-140 also targets BMP2 but in a different position of the 3′-UTR region and is associated with increased BMP2 expression (120). Notably, the levels of synovial miR-140 were significantly reduced in the patients with OA and were negatively correlated with OA severity compared to controls (120, 122). Furthermore, after arthroscopic debridement, the levels of these miRNAs and BMP2 were restored (120), suggesting miR-22 and miR-140 play a role in the development of OA by regulating BMP-2. It has also been shown that BMP targeting miRNAs’ dysregulation is associated with the pathogenesis of RA. It has been demonstrated that sEVs derived from fibroblast-like synoviocytes with elevated levels of miR-486-5p promoted osteoblast differentiation and proliferation by repressing Tob1, thus activating the BMP/Smad signaling pathway, alleviating the severity of RA in the CIA model (123).

On the other hand, TGF-β has been implicated in cartilage ECM production and maintenance, specifically by increasing COL2A1, perlecan, fibronectin, and hyaluronan (124, 125). Furthermore, TGF-β also has anti-inflammatory functions, counteracting IL1b and IL-6 mediated inflammation in the joint (124, 125). Importantly, several miRNAs target different proteins of these pathways, which has been reviewed elsewhere (126). It has been shown that miR-455-3p promotes TGF-β/Smad signaling in chondrocytes and inhibits cartilage degeneration by directly suppressing PAK2, a kinase that inhibits TGF-β signaling. Accordingly, the miR-455-3p levels were decreased, and both PAK2 and phospho-PAK2 were increased in OA cartilage compared with control cartilage. Moreover, miR-455-3p KO mice displayed significant degeneration of the knee cartilage (127). In OA cartilage, miR-150-5p is overexpressed. It has been shown that miR-140-5p directly targets TGF-b3 signaling by altering the expression of TGF-b3 and Smad-3 in mandibular condylar chondrocytes, thus having a role in the regulation of mandibular cartilage homeostasis and development (128). Furthermore, this miRNA is increased in the cartilage of OA patients compared to control cartilage from femoral neck fracture patients, where it suppresses the Smad2/3 pathway, a process that promotes cartilage destruction and the progression of the disease (129). Using miR-140-null mice, which showed different changes related to OA such as fibrillation of articular cartilage, Miyaki and collaborators demonstrated that miR-140 regulates cartilage development and homeostasis (113). Interestingly, miR-140 knockout mice presented proteoglycan loss and fibrillation of articular cartilage emulating age-related OA. On the contrary, transgenic mice overexpressing miR-140 in cartilage were resistant to antigen-induced arthritis. Another miRNA involved in TGF-β signaling modulation is miR-125-5p, which downregulates the Smad2 expression and leads to the dysfunction of TGF-β signaling. Noteworthy, the circular ribonucleic acids (circRNAs), CircCDK14, which is down-regulated in the joint wearing position, regulates metabolism, inhibits apoptosis, and promotes chondrocyte proliferation by miR-125a-5p sponging (130). Taking together, studying miRNA dysregulation in OD and the underlying mechanisms could provide new insights towards more effective treatments. At the same time, TGF-β exerts an anabolic repairing response on articular cartilage. On the other hand, proinflammatory cytokines such as IL-1β and TNF-α which exert a strong catabolic effect (131). As follows, the balance between TGF-β and the IL-1β or TNF-α signaling pathways is a critical regulator of articular cartilage homeostasis (131), thereby its disruption contributes to the pathogenesis of OA.

In OA, NF-κB signaling orchestrates chondrocyte catabolism, survival, and synovial inflammation. Growing evidence suggests that miRNAs targeting either matrix-degrading enzymes or components of the NF-κB pathway can suppress chondrocyte catalytic activity. While some miRNAs such as miR-138 and miR-9 directly suppress the NF-κB subunits p65 or p105/50 (132, 133), others like miR-210, miR-26a/b, miR-93, miR149, and miR-146a act indirectly by targeting upstream regulators of NF-κB (134) such as death receptor 6 (DR6), KPNA3, Toll-like receptor 4 (TLR4), TAK1, and TNF-receptor associated factor 6 (TRAF6)/interleukin-1 receptor-associated kinase 1 (IRAK1). Additionally, synovial inflammation in the context of OA or osteoblastogenesis is associated with miR-146/miR-155/miR-218/miR-135, among others (135–137).

In RA, miRNA dysregulation is implicated in the activation of multiple cytokine-signaling pathways that leads to synovial tissue lesions and dysregulation of immune cells, thereby contributing to pathogenesis (139). Many studies have demonstrated that miR-16, miR-146a, miR-155, and miR-223 present an increased expression level in synovial fluid of RA patients. Moreover, inflamed joints of RA patients show an increased expression of miR-133a, miR-142-3p, miR-142-5p, miR-146a, miR-155, miR-203, miR-221, miR-222, miR223 (103, 107, 140, 141). On the other hand, the expression of miR-124a and miR-34a is decreased in the context of RA (142, 143). Furthermore, miR-181a, miR-17–92 overexpression enhances the inflammation, while upregulation of miR-146a and miR-573 suppresses the autoimmunity (144). Although several miRNAs related to inflammation are dysregulated in RA, miR-146a appears to be essential in controlling the inflammation. miR-146a targets TNF-α/TNF receptor-associated factor 6 (TRAF6) and IL-1 receptor-associated kinase 1 (IRAK1), elevating TNF-α production through TRAF6/IRAK1 mediated pathway [Reviewed in (126, 145)]. miR-146a is also able to regulate genes such as FAF1, IRAK2, FADD, IRF-5, Stat-1, and PTC-1 (146), making it a possible therapeutic target for the treatment of RA. Besides miR-146, miR-155 can also stimulate the proinflammatory mediators TNF-α, TLRs, LPS, and IL-1 [Reviewed in (145)]. Upregulation of miR-155 has been observed in synovial tissue, FLS, peripheral and blood mononuclear cells. Supporting a role for targeting miR-155 in RA, miR-155 knockout mice do not develop collagen-induced arthritis (146). Therefore, miR-155 may be a promising therapeutic target for RA.

miRNAs and their levels in plasma and synovial fluids are associated with the occurrence of OD. Therefore they could serve as predictive biomarkers and even as therapeutics targets. Owing to the fact that miRNAs play a crucial role in the maintenance of healthy joints, restoring their balance could be an effective way to treat OA and RA. To accomplish an effective therapeutic strategy, the delivery system is the main barrier that has to be overcome (147). Given that miRNAs are naturally carried by sEVs, they are protected from RNAse degradation and the delivery to target cells is efficient thanks to the integrins and opsonins (147–150).

## miRNA Shuttled by sEVs Derived From MSCs and Their Therapeutic Function on Osteoarticular Diseases

Since MSC-sEVs are natural carriers of therapeutic miRNA, they have arisen as an attractive therapeutic tool to treat several diseases including OD. There are copious amounts of studies reporting the different effects of miRNA transfer *via* sEVs, and their relevance in cell to cell communication. Indeed, miRNAs have gained more attention than proteins or other variety molecules contained in sEVs, due to their regulatory roles in gene expression. Goldie and collaborators demonstrated that the proportion of miRNA is higher in sEVs than in their parent cells (151). Moreover, a profiling study of miRNAs has demonstrated that miRNAs are not randomly packaged into sEVs. Guduric-Fuchs and collaborators have shown that a subset of miRNAs (miR-150, miR-142-3p, and miR-451) are preferentially incorporated in sEVs (152). Although the effects of other sEV cargos cannot be excluded, miRNAs are considered the key functional elements on recipient cells. Several thousand miRNAs have been identified in humans, and their studies have increased in the last decade, moreover miRNAs are frequently deregulated in multiple human diseases which offers many opportunities for diagnosis and treatment for various pathological conditions.

The use of sEVs as a therapeutic treatment for different immune diseases is still challenging, since safety evaluations are still pending. Multiple experiments must be done in large and proper animal models in order to prove their therapeutic efficacy and safety in this area before applying this approach in the clinic. Given that it primarily affects the joints, we suggest that the optimal form of delivery should be intra-articular injection.

Chen and collaborators, have shown that, both *in vitro* and *in vivo*, BM-MSC-sEV enriched in miR-150-5p suppress the expression of MMP14 and VEGF, and decrease the expression levels of IL-β, TNF-α, and TGF-β, resulting in the inhibition of the proliferation and migration of fibroblast-like synoviocytes (FLS) and alleviation of inflammation (153). Similarly, BM-MSC sEV derived miR-320a targets CXCL9 and thereby suppresses FLS activation, migration and invasion in RA (154). Additionally, the overexpression of miR-124a in MSC-sEV significantly increased the expression of apoptosis-related proteins inducing an inhibition on the proliferation, invasion and migration of RA-FLS cells (155).

**Figure 1:**
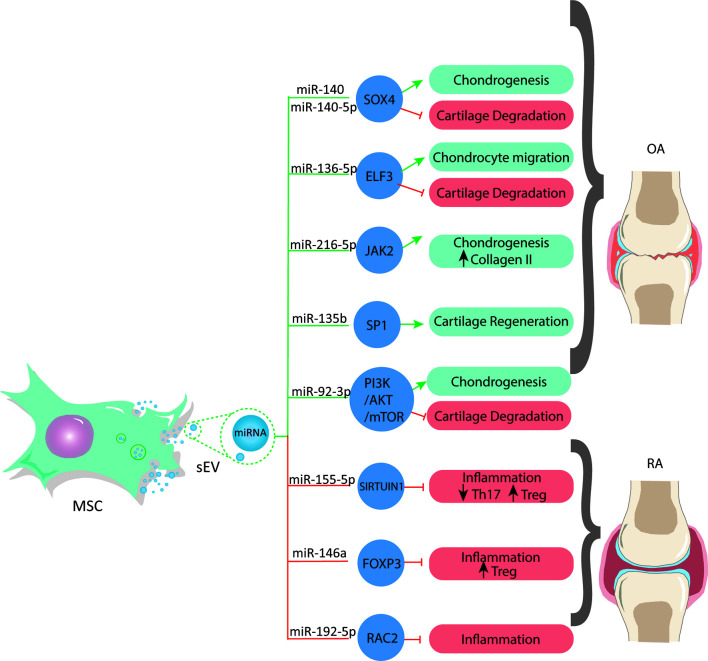
MSCs release sEVs with a miRNAs cargo that regulate gene expression by targeting transcription factors associated to different processes in osteoarticular diseases. These miRNA can be used to develop new and effective therapies for OA and RA.

It has been well documented that miRNAs in MSC-sEVs have a chondroprotective role in OA (156). Illustrating this, MSC-sEVs shuttled miR-92a-3p increases chondrocyte proliferation and the levels of COL9a2 and aggrecan, and effect mediated by targeting noggin3 and Wnt5a while activating the PI3K/AKT/mTOR pathway, thus increasing the levels of [Reviewed in (21)], (88). On the other hand, MSC-sEVs-derived miR-135b stimulates cartilage regeneration by binding to the transcription factor Sp1 (SP1), which regulates apoptosis and proliferation (157). Moreover, miR-140-5p upregulates Sox9 and promotes MSCs chondrogenesis (**Figure 1**). Additionally, recent studies show that sEV-mediated transfer of miR-140 from dendritic cells improves OA *in vitro* by inhibiting proteases associated with cartilage degradative processes in the joint and alleviates the progression of OA in a rat model *in vivo* (158). In contrast, another study reported that miR-155 levels are significantly upregulated in human OA cartilage biopsies and primary chondrocytes stimulated by IL-1ß. Moreover, miR-155 overexpression promotes IL-1ß-induced apoptosis and catabolic activity in chondrocytes *in vitro* (159). Chen et al. reported that MSC-sEV-shuttled miR-136-5p promotes chondrocyte migration *in vitro* and inhibits cartilage degeneration *in vivo* (**Figure 1**) both in human chondrocytes *in vitro* and in mice *in vivo* (160).

On the other hand, the involvement of MSC-sEVs-derived miRNAs in the context of immune modulation has been reported (149). MSC-sEVs are immunologically active, meaning that they can attenuate the immune system through increasing anti-inflammatory cytokines, such as IL-10 and TGF-β and the induction of Tregs, modulating immune activity. Indeed, RNA-depleted sEVs lose their immunosuppressive activity on T-cells (161), demonstrating their pivotal role on MSC-sEVs immunoregulation and therefore their potential use on autoimmune diseases such as RA (75, 80, 83). Indeed, the downregulation of miR-192-5p has been reported in RA patients, and its transfer *via* sEVs derived from BM-MSCs reduced the inflammatory response by downregulating the Ras-related C3 botulinum toxin substrate 2 protein (RAC2) (**Figure 1**), attenuating the severity of the disease in rats (162). It has been reported that sEVs derived from TNFα and IFNγ pretreated-MSCs improve their suppressive activity over T cells (75). This pretreatment was associated with a higher expression of miR-155 and miR-146, two miRNAs involved in activating and inhibiting T cells inflammatory reactions (163). Similarly, miR-155-5p loaded in sEVs derived from LPS-stimulated periodontal ligament stem cells (PDLSCs) inhibited pro-inflammatory Th17 cells favoring their conversion into Treg through inhibition of Sirtuin-1 (Sirt1) (164). Moreover, the therapeutic role of miR-146a-5p contained in MSC-sEVs has been shown *in vivo* in a model of allergic airway inflammation (161). In this study, the authors demonstrated that the miRNA signature of MSC-sEVs was enriched in miR-146a-5p compared to sEVs derived from less immunosuppressive cells such as fibroblasts (161). In addition, miR-146a-5p mimic improves the immunosuppressive capacities of fibroblast sEVs, while miR-146a-5p inhibition impairs the immunosuppressive activity of MSC-sEVs on T-cell proliferation (161). In RA, miR-146a is downregulated, but its upregulation associated with the administration of MSC-sEVs increased the frequency of Treg cell population by increasing the expression of some key autoimmune response genes and their protein products, such as TGFβ, IL-10 and FOXP3 (**Figure 1**), resulting in a beneficial anti-inflammatory response (165, 166). Rong and collaborators showed that the hypoxic pre-treatment of rat BM-MSC (a known method for the improvement of the therapeutic properties of MSCs [Reviewed in (167)]) promotes the release of miR-216a-5p enriched sEVs that target JAK2 in chondrocytes, resulting in an increase in chondrocyte proliferation and migration, while inhibiting their apoptosis. The miR-216a-5p enriched sEVs also reduced ECM degradation through the inhibition of MMP expression and increasing COL-II expression levels (168).

In summary, several miRNAs are known to be associated with different processes relevant to OD (169), such as inflammation (miR-22, miR-320) (105, 110), extracellular matrix synthesis (miR-148a, miR-27, miR-218) (170, 171) and chondrocyte proliferation. Additionally, several miRNAs have been shown to be involved in processes associated with MSCs differentiation into chondrocytes (miR-19a, miR-410) (172, 173), and processes such as chondrocyte hypertrophy (miR-381, miR-140) (174, 175), apoptosis and autophagy (miR-30b) (176) (**Table 1**). The therapeutic potential of miRNAs both in degenerative diseases such as OA and autoimmune diseases such as RA is very promising, and their delivery through sEVs greatly facilitates escalation to later-stage clinical trials. Still, more work needs to be done concerning the full effect of miRNAs both in target cells and other types of cells to assess the safety of the therapeutic application of miRNAs.

**Table 1:** Summary of the literature reporting the role of miRNAs in OD.

miRNA	Context	Target cell	Effect on the target cell	Mechanism of action	Reference
miR-92a-3p	sEVs from miR-92-3p-overexpressing MSCs; OA	Chondrocytes	Enhancement of chondrogenesis and suppression of cartilage degradation	Targeting the PI3K/AKT/mTOR pathway	88
miR-135b	sEVs from TGF-β1-stimulated MSCs	Chondrocytes	Cartilage regeneration	Binding to transcription factor (SP1)	157
miR-22	OA; inflammation	Chondrocytes	Decrease inflammation and ECM degradation	Targeting the PPARα and BMP-7 signaling pathway	105
miR-140	OA; MSC-sEVs	Chondrocytes; MSCs;	Inhibition of cartilage degradation; suppression of chondrocytes hypertrophy; Promotion of chondrogenesis	Suppression of the expression of cartilage degrading enzymes; controlling the BMPs signaling pathway; Upregulation of Sox9	158; 175; 177
miR-320	Cartilage homeostasis	Chondrocytes	Regulation of chondrogenesis	Targeting the expression of MMP-13	110
miR-27	OA	Chondrocytes	Decreasement of inflammation	Inhibition of the NF-κB pathway	170
miR-149	OA inflammation	Chondrocytes	Suppression of chondrocyte inflammatory response	Downregulation of the TAK1/NF-κB pathway	135
miR-19a	OA	Chondrocytes	Promotion of cell viability and migration	Upregulation of Sox9 *via* the/NF-κB pathway	173
miR-410	OA	MSCs	Chondrogenic differentiation	Targeting the Wnt signaling pathway	172
miR-381	OA pathogenesis	Chondrocytes	Chondrocyte hypertrophy	Targeting histone deacetylase 4 (HDAC4)	174
miR-125b	OA	Chondrocytes	ECM degradation	Targeting of ECM-degrading enzyme ADAMTS-4	178
miR-558	OA	Chondrocytes	Cartilage homeostasis	Inhibiting COX-2 and IL-1β-induced catabolic effects	178
miR-9	OA	Chondrocytes	Suppression of apoptosis and promotion of cell proliferation	Binding to NF-kB1	132
miR-138	OA	Chondrocytes	Decrease in the chondrocyte inflammatory response	Suppressing the protein levels of p65, COX-2 and IL6	133
miR-136-5p	OA; MSC-sEVs	Chondrocytes	Increase in chondrocyte migration and decrease in cartilage degradation	Inhibiting the expression of ELF3	160
miR-153	OD	MSCs	Decrease in osteogenic differentiation	Interacting with bone morphogenetic protein receptor type II (BMPR2)	134
miR-194	Bone homeostasis	MSCs	Increase in osteogenic differentiation	Suppressing STAT1	179
miR-216a	OD; MSC-sEVs	MSCs; chondrocytes	Increase in osteogenic differentiation; increase in chondrocyte proliferation and migration	Downregulation of c-Cbl; inhibiting JAK2	180; 168
miR-126a-5p	OA	Chondrocytes	Reduction in ECM degradation	Increasing expression of collagen II and decreasing expression of MMP	168
miR-146a	RA; MSC-sEVs	Tregs	Increase in anti-inflammatory response	Increasing the expression of FOXP3	83

## Concluding Remarks

As mentioned in the previous sections, MSC-sEVs arise as a potential cell-free based therapy that can reduce the risks associated with MSC. Strikingly, several reports show that MSC-sEVs mimic the biological effects of MSCs. Therefore, MSC-sEVs represent a hopeful alternative to MSC therapy.

The main functional components of MSC-sEVs are miRNAs, which can regulate the expression of multiple target genes and participate in various cell signaling processes. The miRNA profile of MSC-sEVs is associated with their effect. Although there are tools to identify miRNAs in sEVs, the principal target genes of sEVs derived miRNAs remain unspecified. However, this work summarizes some of the miRNAs involved in OD pathogenesis and some of the miRNAs that mediate the therapeutic effects of sEVs in OD. These miRNA could be considered as promising candidates to use for effective treatment of these diseases. Further studies in this field are required to develop MSC-sEVs therapeutics based on miRNA delivery for autoimmune/inflammatory and degenerative diseases. Furthermore, delving into the role of miRNAs in the pathogenesis of disease, would also improve therapeutic strategies that can restore their normal levels, because not all miRNAs have beneficial effects. In this context it is also important to study the regulation of miRNAs and their biological functions, and also increase the knowledge of other non-coding RNAs that can be involved in OD. On the other hand, studies on the enrichment of sEVs in beneficial miRNAs and/or other non-coding RNAs that regulate disease-promoting miRNAs and evaluating strategies for the targeted delivery of sEVs to particular cell types to increase efficiency remain one of the following challenges.

## Author Contributions

EL-B and MA wrote the main part of the manuscript with inputs from CH, FB-B, AO, CG, FG, CP, NL-C, GM, RE-V, and FD. PL-C and AV-L design the original idea of the review and critical review the manuscript. All authors contributed to the article and approved the submitted version.

## Funding

This review was supported by grants from the Agencia Nacional de Investigation y Desarrollo (ANID) from Chile through the Fondecyt Regular program grant number 1211353 PI PL-C. We thank the “Agence Nationale de Recherche” for the ANR METAB-OA (ANR-20-CE18-0014) and PRI-mitoMir.

## Conflict of Interest

The authors declare that the research was conducted in the absence of any commercial or financial relationships that could be construed as a potential conflict of interest.

## Publisher’s Note

All claims expressed in this article are solely those of the authors and do not necessarily represent those of their affiliated organizations, or those of the publisher, the editors and the reviewers. Any product that may be evaluated in this article, or claim that may be made by its manufacturer, is not guaranteed or endorsed by the publisher.
